# Life cycle assessment and sustainable engineering in the context of near net shape grown components: striving towards a sustainable way of future production

**DOI:** 10.1186/s12302-017-0125-x

**Published:** 2017-10-20

**Authors:** Christoph Kämpfer, Thomas-Benjamin Seiler, Anna-Lena Beger, Georg Jacobs, Manuel Löwer, Franziska Moser, Julia Reimer, Martin Trautz, Björn Usadel, Alexandra Wormit, Henner Hollert

**Affiliations:** 1 Institute for Environmental Research, RWTH Aachen University, Worringerweg 1, 52074 Aachen, Germany; 2 Chair and Institute for Engineering Design, RWTH Aachen University, Steinbachstr. 54 B, 52074 Aachen, Germany; 3 Chair of Structures and Structural Design, RWTH Aachen University, Schinkelstr. 1, 52062 Aachen, Germany; 4 Institute for Biology I, RWTH Aachen University, Worringer Weg 3, 52074 Aachen, Germany

**Keywords:** Life cycle assessment, Environmental impact assessment, Ecotoxicology, Bio-based production, Bioeconomy, Design with nature, Sustainable construction materials

## Abstract

Technical product harvesting (TEPHA) is a newly developing interdisciplinary approach in which bio-based production is investigated from a technical and ecological perspective. Society‘s demand for ecologically produced and sustainably operable goods is a key driver for the substitution of conventional materials like metals or plastics through bio-based alternatives. Technical product harvesting of near net shape grown components describes the use of suitable biomass for the production of technical products through influencing the natural shape of plants during their growth period. The use of natural materials may show positive effects on the amount of non-renewable resource consumption. This also increases the product recyclability at the end of its life cycle. Furthermore, through the near net shape growth of biomass, production steps can be reduced. As a consequence such approaches may save energy and the needed resources like crude oil, coal or gas. The derived near net shape grown components are not only considered beneficial from an environmental point of view. They can also have mechanical advantages through an intrinsic topology optimization in contrast to common natural materials, which are influenced in their shape after harvesting. In order to prove these benefits a comprehensive, interdisciplinary scientific strategy is needed. Here, both mechanical investigations and life cycle assessment as a method of environmental evaluation are used.

## Background

Since the midst of the twentieth century increasing negative influences on the environment were observed, caused by the progressive industrial development of mankind. First, the global warming is attributable to increasing greenhouse gases emissions caused by the strong economic and population growth, which never has been as strong as in the last decades [1]. Second, biodiversity and the fulfilment of ecosystem services is highly threatened by the increasing attempts to satisfy the need for food, fresh water, timber, fibre and fuel [2]. These activities are believed to have the potential to cause drastic environmental changes. As a consequence, an emerging environmental awareness developed in the scientific community [3, 4]. As one scientific response, the planetary boundaries framework was introduced in 2009 [5, 6] and revised in 2015 [7]. Within the planetary boundaries framework, a safe operating space based on intrinsic biophysical processes responsible for stability on Earth gives guidance for further human acting and development to preserve this state. Local and regional boundaries evolved to limit the emission to the environment and extractions from the environment, respectively, as well as impacts related to land use and ecosystems. Within the list of boundaries, climate change and biosphere integrity are identified as the two core boundaries that can threaten Earth’s stable state. Beside these, e.g. biodiversity loss and chemical pollution are considered as further important boundaries [7].

Modern industrial production creates high quality products, but is also related to environmental damage as remarked within the planetary boundaries framework. Plastics, as widely used materials in numerous products, contain harmful substances and are produced in highly energy-intensive processes and therefore contribute to problems like chemical pollution of the environment and climate change. Bisphenol A (BPA) is one plastic contaminant of major concern. It was originally synthesised as a chemical oestrogen [8]. Nowadays, BPA is ubiquitous, due to its various fields of application, including monomer for the production of polycarbonate plastics and epoxid resin, metal can lining, plastic material of or plasticizer in consumer products, such as toys and drinking containers, as well as its use in medical equipment. As a result, many occasions exist for humans to get into contact with this substance [9, 10]. BPA concentrations up to 1.49 ng/mL were measured in human serum [11]. In many water bodies, concentrations of even up to 12–43 µg/L can be observed [12]. For aquatic organisms, BPA results in feminisation even at low concentrations, as had been exemplarily shown for *Xenopus laevis* tadpoles [13]. Furthermore, aquatic species accumulate BPA, mainly in the liver but also at lower levels in muscle tissue [14].

## Recent scientific approaches to address these issues

In light of the aforementioned negative side effects of industrial production, which indicate the need for innovative and environmentally benign solutions in future development of consumer and investment goods, research activities like “Green Engineering” [15], “Green Chemistry” [16] and “Green Toxicology” [17] have evolved in the past years and are aiming at more sustainability.

In order to facilitate the transition towards a more sustainable future through science and technology, Green Engineering provides a design framework for new materials, products, processes and systems. The two main ideas within Green Engineering are (1) the consideration of the whole life cycle, and (2) the idea of inherency. Hence, when thinking about the construction of products in a sustainable way, the life cycle of the product itself as well as the life cycles of all material and energy inputs have to be considered. Thus, the environmental performance of the product itself cannot be distorted while shifting, e.g. toxic impacts into upstream processes. The second way is to shift the inherent properties of the product towards harmlessness. In case of unfavourable circumstances, the benign, inherent properties of a product will be a protection against severe consequences [15].

Similar to the approach of Green Engineering, substances can be designed according to the paradigm of Green Chemistry. During both the production process and the use phase, as little risk as possible to human health or the environment should be caused [16].

To facilitate the production of environmentally friendly substances, monitoring methods and real-time-analysis in early stages of the product development have to be established. The Green Toxicology concept is one way to support this process by delivering a framework for high-throughput and high-content methods to identify whether a substance is worth further development from an ecotoxicologist’s point of view. In consequence, unintended effects on human health and the environment, or the very expensive “phase out” at a later stage of the product development, can be avoided [17].

Herein, we want to present and discuss the idea of near net shape growth of plant material. This means a new framework in which a novel way of construction with plant material is combined with environmental footprint analysis to a holistic concept of sustainable, bio-based production. Near net shape growth means to utilise plants as technical materials and find out to what extent it is possible to influence the growth of a living plant in terms of imposing it into wanted directions and shapes. In particular, it is part of the concept to investigate if the shape of plants can be manipulated to let them grow into the shape of the final product as far as possible. Thus, it is assumed to not only create more eco-friendly products for a better eco-balance of the material, but additionally it would be possible to have less manufacturing steps. Consequently resources can be saved during the manufacturing process.

## Technical development combined with life cycle assessment to reduce the environmental footprint of near net shaped components

### Technical product harvesting (TEPHA): an innovative way of production

Against the background of global warming, the limited resources, and the increasing ecological awareness in society as well as research efforts in the field of sustainability, growing near net shape components from renewable materials has the potential to lessen the environmental impact of the product. The development of this technical product harvesting highly benefits from an interdisciplinary viewpoint, which considers the technical implementation as well as aspects of sustainability. Thus, a unilateral perspective either from the technical or the environmental side can be prevented.

One major goal is to fully exploit the potential of natural materials and shapes for applications in consumer or investment goods. Products are to be identified, where the change from conventional materials like plastics or metal to a renewable material is reasonable. In parallel, new ways to utilise plants as technical materials are identified. The shape of the final product is approximated as far as possible already during plant growth. By using natural materials, a better eco-balance is expected. In addition, such an approach will significantly reduce manufacturing steps. As a consequence resources could be saved during production. Furthermore, it is expected that plants which are being influenced during their growth will go through a natural topology optimisation. Grown structures can lead to mechanically more stable products, compared to plants or parts of plants being shaped under, e.g. pressure and heat after they have been harvested [18]. Because the approach is inherently interdisciplinary, a new type of construction with natural materials and an integrated concept of construction and environmental assessment is strived for, which can be seen as an innovative approach. Accordingly, in order to test the feasibility of this approach from a technical as well as an ecological perspective, appropriate studies cannot only be carried out by one discipline, but only through the already mentioned close interdisciplinary cooperation.

### First steps towards technical product harvesting: the product database as theoretical basis

**Fig. 1:**
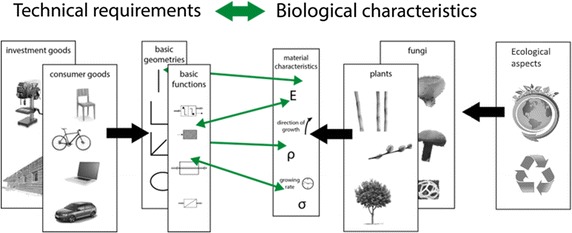
Systematic approach to match technical requirements with biological characteristics (Adapted from [22]. The permission of the copyright holder for the use and adaptation of the figure exists)

To systematically approach the idea and to ensure not to overlook any potential solutions, in the TEPHA framework, the different products are clustered and broken down to their elementary functions. Possible products and use cases have to be analysed from an engineering perspective, categorised and grouped. As basis for a resulting database, this outcome should comprise information about basic product functions as well as required mechanical, geometrical and material/substantial properties. In parallel, data about suitable plants and organisms is collected and systematically structured. As a first approach, only plants with woody-type structures were taken into consideration. They are characterised by lignification increasing the stiffness and strength of wood [19], which makes it usable for technical applications. Due to the wide use of wood, a large number of woody plants are well investigated and thus mechanical data are available in sufficient quantity and quality. These data were, for instance, composed by Wagenführ [20]. In principle, all plants are possible candidates for the database. Due to the high number of plant species of about 350,000 [21] a coverage as complete as possible will be strived for over a longer period. This will be approached by further literature work and experimental measurements if appropriate data are not available. The data is analysed to find matching features that allow biological ways to reproduce the required technical properties (Fig. 1). Since it is not necessarily true that products made of natural materials are more sustainable compared to their conventional alternatives, the most important environmental aspects connected to the cultivation of plants have to be identified and inserted into the database as well.

The aim is to provide a tool with which a straightforward selection of suitable plants for the intended purpose is possible. It is necessary to clarify which aspects have to be integrated into the database, and how they should be structured. In the further development these fundamental considerations will provide the basis for practical implementation of the near net shaped growth. For the successful realisation in practice, a profound knowledge about the mechanical requirements of products, the mechanical properties of the plants as well as the related environmental aspects is necessary. The database will provide this knowledge.

### Implementation of the theoretical approach into practice

#### Technical and mechanical aspects of the TEPHA approach

In order to show the potential of the approach and analyse its applicability, a variety of proof of concept studies have to be conducted. Since this cannot be carried out for all potential organisms, bamboo is used as an exemplary plant species. Bamboo seems to be an advantageous plant species, since it is already utilised in building, construction for plywood and composites [22]. Most importantly, bamboos belong to the fastest growing species in plant kingdom. For example, the bamboo species *Dendrocalamus giganteus* grows within one growth period of several months to its full length of up to 35 m with a diameter of around 30 cm. Subsequently, the culms lignify and incorporate silica resulting in the typical hard and strong bamboo material [23]. The woody stems exceed tree wood, brick or concrete in terms of compressive strength and can compete with steel when compared for tensile strength [24]. Finally, the outer surface of the hollow stem with longitudinal fibres and intersections shows the most favourable properties in terms of hardness [22, 24].

The feasibility studies include systematic growth manipulations and aim at determining the boundaries and benefits of growth manipulations. For this purpose, it has to be figured out how growth manipulation shapes have to be designed to allow for systematic growth observations, and form manipulations as accurate as possible. Moreover, the studies are designed to result in a bamboo seat that can then be compared to a conventionally produced plastic (polypropylene, polycarbonate) seat. Both test series are complemented by a set of comparative experiments to contrast the material properties (e.g. pressure resistance) of growth manipulation to conventionally grown organisms.

This investigation facilitates the evaluation of the novel approach and offers the possibility to quantify the impact of natural topology optimisation.

When a new technology is applied in industrial scale, an in-depth understanding of the interactions with the environment is necessary. Therefore, environmental investigations are needed which consider a broad range of environmental impacts [25].

#### Life cycle assessment in the context of technical product harvesting as a tool for the prospective evaluation of possible environmental impacts

**Fig. 2:**
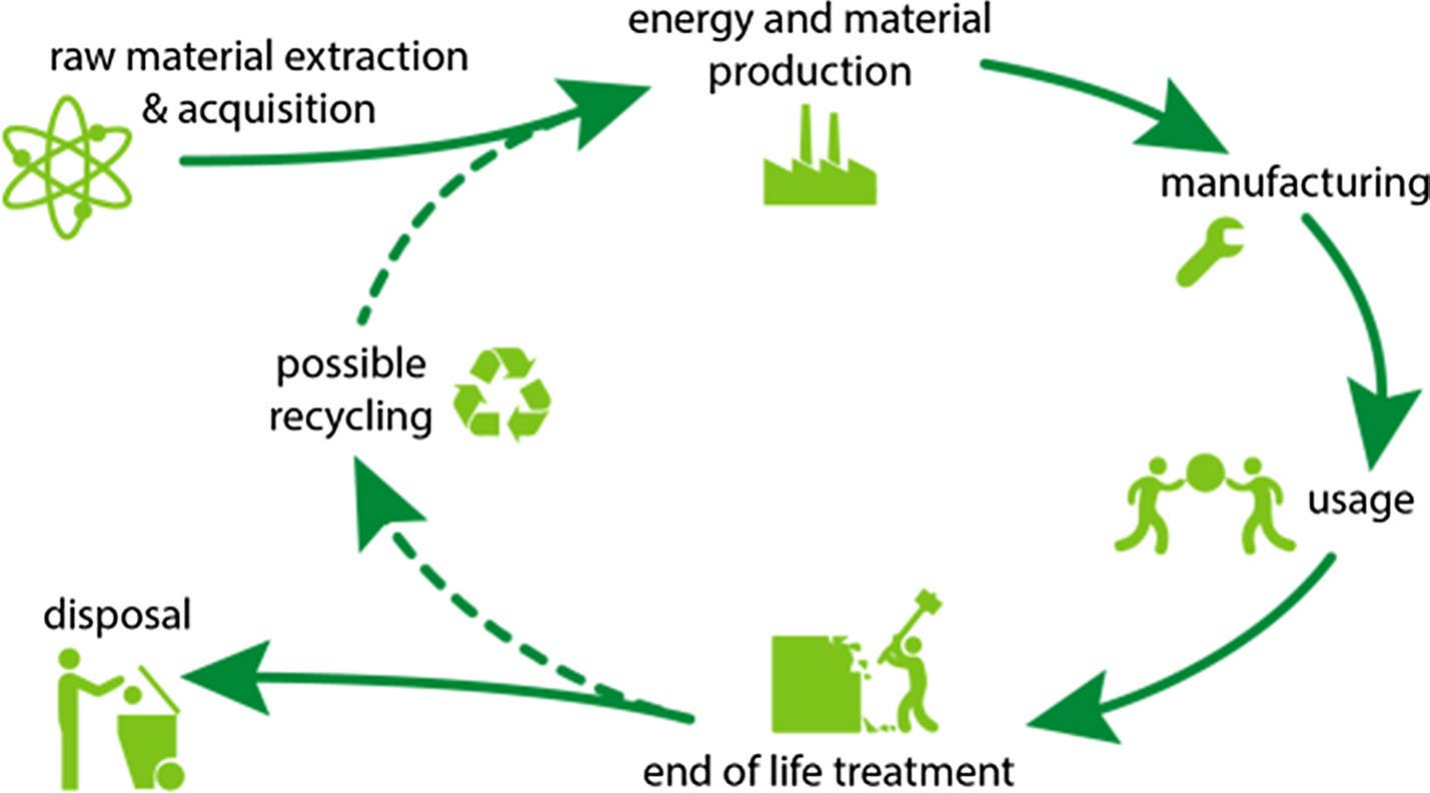
Fundamental stages within a product life cycle [22] (The permission of the copyright holder for the use of the figure exists)

The basic idea of a life cycle assessment (LCA) is to set up a holistic environmental analysis of all substance and material flows throughout the whole life cycle of a given product. The analysis comprises every step from raw material acquisition, production process, transport, use, reuse, recycling, to disposal (Fig. 2). This holistic approach avoids a narrow perspective of the environmental burden of products [26]. If only the product life stage was investigated, an inaccurate weighting of the environmental impacts with regard to the whole life cycle can occur. “For instance, making a car out of aluminium instead of steel means that its gasoline consumption is reduced, but the production of aluminium requires more energy than that of steel. Only when all these facts are taken into account can it be judged whether a car made of aluminium is truly more environmentally friendly than one made of steel” [27].

One crucial aspect of near net shape growth is to promote the development of products showing lower environmentally hazardous potential than their conventional equivalents. To assess whether this innovative way of production is more sustainable, life cycle assessment should be started during the first steps of product development. Thus, possible environmental problems associated with this new type of production can be identified at an early stage and taken into account in the further technical product development. Within the proof of concept study using bamboo as exemplary plant, an LCA of the near net shape grown components as well as of conventionally manufactured equivalents has to be conducted.

**Fig. 3:**
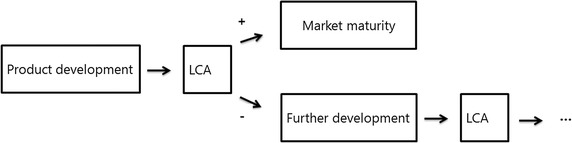
LCA to direct the development of new products towards the most sustainable solution

The goal of this LCA study is to quantify the environmental impacts occurring in the entire life cycle of a near net shape grown seat (“cradle to grave”). Since the environmental benefits of near net shape growth of technical components have to be shown a comparison with conventional materials like plastics as well as conventionally shaped bamboo using heat is inevitable. The most important steps in terms of environmental impacts (hot-spots) within the life cycle can be identified. If severe impacts are obtained, these results will guide the further product development to reduce the environmental load (Fig. 3). This will deliver starting points for the optimisation of the envisaged seat aiming at the reduction of adverse impacts on the environment and human health. Results will help to gain an in-depth understanding of the interactions between the product life cycle and the environment, to avoid possible undesired effects when the product becomes introduced to the market.

As usual for LCA [26] also within the TEPHA framework, the proven green characteristics could then be used for communication towards regulators, e.g. regarding climate change impacts and as a unique selling point, if the new bio-based product turns out to be more environmentally friendly compared to a conventional alternative.

Within this study, the resource acquisition, the production process, distribution, use, and end of life treatment of a seat made of near net shape grown bamboo-based components as well as a conventional seat made of plastics has to be analysed.

The environmental impacts of the near net shape grown bamboo seat are compared with a conventional seat made of polypropylene. In order to test the suitability of the bamboo as a material for this application, further investigations based on the inputs and outputs of the respective product system have to be carried out.

Bamboo is not native to Germany. Here, it is investigated to which extent the climate conditions affect the factors included in the LCA inputs. Since most bamboo is grown in China or Central America, the LCA study also analyses the case where the bamboo culms are shipped to Europe. This investigation gives insights whether the transport from bamboo native countries with its favourable environmental conditions or the management measures in Europe (e.g. watering) cover a greater share of the overall environmental performance.

A characteristic feature of the TEPHA concept is shaping the outgrowth of bamboo by an external shape. However, this shape has to be produced and is, in consequence, not free from environmental impacts. Thus, during the development of the TEPHA approach, it is necessary to find out the best material for the shape in order to fulfil its function and at the same time to minimise negative environmental impacts. The question, therefore, is whether classical plastics based on fossil resources are to be preferred or rather bio-based plastics, which are based on renewable resources.

Moreover, bamboo culms are already shaped for various products using heat. Here, it is crucial to find out if the developed TEPHA approach is also beneficial from an environmental point of view if no shape is needed and the bamboo culm is shaped under energy use.

To make an informed statement about which material and which process for the construction of a seat is the most sustainable choice the results of the entire study are summed up and evaluated critically.

## Conclusion

Near net shape grown production could be an approach to mitigate the environmental impacts associated with the production processes as well as the materials used for consumer and investment goods. The database is a fundamental element of the TEPHA approach to identify plants with the right properties with regard to mechanical and ecological suitability. Here, the most important part is to build an extensive database helping to choose the suitable plant for each application. In order to further develop this approach, strong interdisciplinary collaboration is needed, and the approach has to be implemented theoretically sound but also with a strong focus on the applicability.

## Abbreviations

TEPHA: technical product harvesting
LCA: life cycle assessment
BPA: bisphenol A
